# Supplementary material for: Old carbon routed from land to the atmosphere by global river systems
Source: Nature. 2025 Jun 4;642(8066):105–11. doi: 10.1038/s41586-025-09023-w (PMC12137138; doi:10.1038/s41586-025-09023-w)
Supplement: Supplementary file 1 — This file contains Supplementary Discussion, Supplementary Figs. 1–9, Supplementary Tables 2, 4 and 5 and Supplementary References. [file 41586_2025_9023_MOESM1_ESM.pdf]

nature portfolio
https://doi.org/10.1038/s41586-025-09023-w

Supplementary information

Old carbon routed from land to the atmosphere by global river systems

In the format provided by the
authors and unedited

Supplementary Information

Old carbon routed from land to the atmosphere by global river systems

Joshua F. Dean, Gemma Coxon, Yanchen Zheng, Jack Bishop, Mark H. Garnett, David Bastviken, Valier Galy, Robert G. M. Spencer, Suzanne E. Tank, Edward T. Tipper, Jorien E. Vonk, Marcus B. Wallin, Liwei Zhang, Chris D. Evans, & Robert G. Hilton.

Contents

Pg. 1 – Supplementary discussion

S1. Summary of global ^{14}C patterns in river DIC, CO_2 and CH_4

S2. $F^{14}C$ equilibrium between CO_2 and total DIC

S3. Bayesian isotope mixing model

S4. Upscaling limitations

Pg. 6 – Supplementary Figures (S1-S9)

Pg. 13 – Supplementary Tables (S1-S5)

Pg. 16 – References

Supplementary discussion

S1. Summary of global ^{14}C patterns in river DIC, CO_2 and CH_4

The dataset has good spatial coverage (Fig. 1A) but there is a bias towards the northern hemisphere (which represents 68% of global land area), with only 9% of samples (n = 108) collected from southern hemisphere locations. There is generally good agreement between the proportions of the global landmass underlain by the lithologies and in the biomes defined in this study (Fig. S4). There is a slight bias in the database towards igneous lithologies which tended to have higher (younger) $F^{14}C$ values (Fig. 2B). For biomes, most proportions agreed within ± 10%. The most under-represented biomes were tropical grasslands and shrublands (-13%) and deserts (-15%). Tropical grasslands and shrublands were the biome with second most depleted (older) ^{14}C values (Fig. 2C), indicating a potential bias towards younger carbon; while deserts tended to be younger and are not very productive systems, so likely contribute relatively little carbon to global river DIC. The most over-represented biomes were temperate conifer & boreal forests (+11%) and temperate and mediterranean broadleaf mixed forests (+19%). The former tended to contain less depleted (younger) ^{14}C and the latter more depleted (older) ^{14}C (Fig. 2C), indicating no clear bias towards older or younger DIC in the database based on biome representativeness.

Our database included direct observations of river DIC (n = 973), CO_2 (n = 197) and CH_4 (n = 25; Fig. 1B; Extended Data Table 1). We averaged observations where locations were sampled more than four times in a single year to reduce repeat sampling bias, whilst retaining a geographic and temporal range of samples (remaining n = 1020; Fig. S5; Extended Data Table 1).

Among all observations, 36% were $F^{14}C$ values > 1. For these observations, it is likely that river emissions were dominated by sources containing carbon fixed into biomass and soils via photosynthesis after 1955 CE – i.e., indicative of annual- to decadal-cycling of atmospheric carbon into terrestrial systems and transferred into rivers. For the remaining 64% of observations where $F^{14}C < 1$, river emissions must have contained carbon fixed from the atmosphere before 1955 CE to explain their lower (older) $F^{14}C$ values. The radiocarbon age calculated from the mean $F^{14}C$ value of all assembled observations was 663 ± 1139 ^{14}C years (median = 395 years; Extended Data Table 1).

Following normalisation of river DIC, CO_2 and CH_4 $F^{14}C$ values to the $^{14}CO_2$ values of the atmosphere during the sampling year, we return $F^{14}C_{atm}$ values[1] (Methods). The main patterns in $F^{14}C_{atm}$ were that catchments ≤ 10 km^2 in size had higher (younger) values ($F^{14}C_{atm}$ = 0.891 ± 0.179, median = 0.955) compared to catchments > 10 km^2 ($F^{14}C_{atm}$ = 0.863 ± 0.150, median = 0.891; Fig. 2A). In terms of biomes (Fig. 2C), the montane grassland and shrubland biome had the oldest carbon emissions (lower $F^{14}C_{atm}$ values; 0.703 ± 0.144, median = 0.735), whereas the temperate grassland and shrubland ($F^{14}C_{atm}$ values of 0.949 ± 0.127, median = 1.005), tundra ($F^{14}C_{atm}$ = 0.929 ± 0.121, median = 0.971), and temperate conifer and boreal forest ($F^{14}C_{atm}$ = 0.908 ± 0.142, median = 0.963) biomes had the youngest carbon emissions. The ubiquity of old (low) $F^{14}C_{atm}$ values across all biomes is clear despite disparities in the number of observations and carbon forms.

Another important spatial variable was lithology (Fig. 2B). River reaches underlain by metamorphic lithology had younger carbon emissions ($F^{14}C_{atm}$ values of 0.957 ± 0.092, median = 0.983) than those underlain by igneous lithology (0.903 ± 0.156, median = 0.959) and sedimentary lithology including carbonates (0.848 ± 0.159, median = 0.873). These differences indicate that lithology, via chemical and physical weathering of bedrock and soils is an important control on the age of river carbon emissions. DIC derived from carbonate minerals ($CaCO_3$) will lower $F^{14}C_{atm}$ values. Following carbonic acid (H_2CO_3) weathering of $CaCO_3$, the DIC produced can contribute petrogenic carbon (^{14}C-dead, i.e., contains no measurable ^{14}C because the material is old enough that ^{14}C has radioactively decayed to levels below detection) to the river degassing flux because of the observed isotopic equilibration of DIC species (Supplementary Information S2). The H_2CO_3 weathering of $CaCO_3$ should produce DIC and CO_2 with $F^{14}C$ = 0.5 [2], or indeed lower if sulfuric acid or rock-derived organic carbon derived CO_2 are involved[3]. In addition, weathering of shales and sedimentary rocks containing rock organic matter could contribute ^{14}C-depleted DIC.

S2. $F^{14}C$ equilibrium between CO_2 and total DIC

We assembled a subset of paired DIC and CO_2 observations which suggest these two inorganic carbon pools have very similar $F^{14}C$ values (within 0.02) for the most common pH ranges of natural waters (Fig. S1; Table S3). Given that the $F^{14}C$ notation corrects for stable isotope fractionation[4], this supports the assumption that CO_2 is in isotopic equilibrium with the total DIC pool (i.e., HCO_3^-). As such, our DIC, CO_2 and CH_4 dataset provide crucial insight on the age of CO_2 emissions from river surfaces at the time and location of sample collection.

Considering this in more detail, we note that after removal of repeated sampling locations (Fig. S5), the database is dominated by $F^{14}C$-DIC measurements (n = 884) compared to CO_2 (n = 117; Extended Data Table 1). CO_2 is a component of the total DIC pool, with the total river

DIC concentration defined as the sum of CO_2, bicarbonate ion (HCO_3^-) and carbonate ion (CO_3^{2-}):

$$DIC = CO_2 + HCO_3^- + CO_3^{2-} \quad (S1)$$

The proportion of CO_2 relative to the other carbon species in DIC (HCO_3^- and CO_3^{2-}) is controlled by pH through the set of carbonate equilibria reactions which can be simplified as:

$$CO_2 + H_2O \leftrightarrow H^+ + HCO_3^- \leftrightarrow H^+ + CO_3^{2-} \quad (S2)$$

For illustrative purposes here, CO_2SYS [5] can be used to calculate the DIC species in a solution with concentrations of 500-1000 µmol L^{-1} DIC, in the pH range 7-8 which typifies most streams and rivers. The proportion of CO_2 in the DIC pool would range from ~20 % to 2 %, respectively, while the CO_3^{2-} ion content is negligible. While considered relatively slow in terms of solution chemistry, the equilibration of CO_2 and HCO_3^- in solution is within the order of 20-200 s [6,7], which is effectively instantaneous when considering typical water residence times in catchments varies on the order of days or longer[8]. Isotopic equilibrium between CO_2 and HCO_3^- has been shown to be reached over ~1-2 hours in laboratory experiments[9]. Isotopic equilibrium is thus still fast compared to most hydrological flow path lengths, but not instantaneous. As such, it has been suggested that CO_2 may equilibrate more quickly with the atmosphere before translating this isotopic signature to the total DIC pool through isotopic exchange[10,11]. If the source of exchanged CO_2 is considered to be respiration or the atmosphere, this 1-2 hour lag could mean river CO_2 is biased towards younger (higher) $F^{14}C$ values relative to the total DIC pool. Unfortunately, only a few paired measurements of total DIC and river CO_2 have been made to assess this.

We present a new set of analyses which suggest there is only a small difference between the $F^{14}C$ values of stream and river CO_2 and the $F^{14}C$ values of the total DIC pool for most natural water pH values (Fig. S1). This minimal differences between $F^{14}C$ in CO_2 versus DIC in our analyses support previous findings from a study that measured the $F^{14}C$ content of CO_2 and DIC in Arctic-boreal lakes[12]. Taken together, this means that $F^{14}C$ from DIC can accurately represent the radiocarbon activity of river CO_2 emissions.

Our new analysis comprises of n = 15 paired CO_2-DIC $F^{14}C$ measurements from 11 distinct sites in Scotland and one in Peru, and is compared with n = 9 paired samples from Arctic-boreal lakes (Table S3). These paired samples were collected across a wide pH range of 4.2-9.4 (Fig. S1). The mean difference between $F^{14}C$ in CO_2 and DIC was 0.02 (0.00-0.13), with greater differences only seen at very low pH not commonly found in natural waters or within our database (Fig. S1; Fig S8). While much larger than the typical analytical uncertainty on $F^{14}C$ measurements, this represents ~2% of the range of $F^{14}C$ values measured in streams and rivers (Fig. 1B).

In the database more broadly, $F^{14}C_{atm}$ was less variable and more similar among CO_2 and DIC pools at pH values below 7 (Fig. S8). At higher pH values, $F^{14}C_{atm}$ was more variable particularly for DIC, indicating more variable inputs including from carbonate weathering which would raise pH. Where lower (older) $F^{14}C_{atm}$-DIC values were seen at higher pH, these were matched by generally lower $F^{14}C_{atm}$-CO_2 values, for example in catchments underlain by sedimentary lithologies (Fig. 2B) and the Montane Grassland and Shrubland biome (Fig. 2C). The trend of lower (older) $F^{14}C_{atm}$ in both CO_2 and DIC as pH increases (Fig. S8), indicates that petrogenic inputs from carbonate weathering can influence vertical CO_2 emissions from

rivers. This trend with pH is more pronounced in DIC, likely because of the sampling bias – CO_2 samples are generally collected in systems where pH is lower because most DIC is present as CO_2 at acidic pH (e.g., ref[13]), whereas DIC sampling is more widely applied as can be seen in the larger number of DIC measurements in the database compared to CO_2.

While the existing number of paired DIC and CO_2 $F^{14}C$ measurements is relatively small, an important future research direction could be to explore the paired isotope dynamics across the full range of conditions which would affect their isotopic equilibrium, such as a wide range of pH, carbon concentrations, and in regions dominated by carbonate or black shale lithologies.

S3. Bayesian isotope mixing model

Alongside the dual end-member mixing approach described in the main text (where petrogenic carbon fluxes are used as a prior in a mixing apportionment; Table 1; Extended Data Fig. 5), we also modelled the potential contributions from three carbon sources without any priors other than $F^{14}C$ ranges for each end-member using a Bayesian isotope mixing model[14,15] (Extended Data Fig. 6). We used the *Simmr* package in R to undertake this modelling. The mixing model is based on a mass balance (Eq. S3), fitting the potential contributions of three carbon sources using Markov chain Monte Carlo modelling. The $F^{14}C$ source values and their standard deviations were propagated through the model, and the *Just Another Gibbs Sampler* code was used for model fitting. When the upper confidence interval of the final model outputs were 1.00 ± 0.10, model convergence was accepted[15]. The food web specific functions, such as preferential source contributions or trophic enrichment factors, were excluded from the *Simmr* runs.

$$F^{14}C_{group} = a \times F^{14}C_{decadal} + b \times F^{14}C_{millennial} + c \times F^{14}C_{petro} \qquad \text{(S3)}$$

$F^{14}C_{group}$ represents the $F^{14}C$ of the DIC, CO_2 or CH_4 from the database grouped either by biome (Extended Data Fig. 6A) or lithology (Extended Data Fig. 6B). $F^{14}C_{group}$ represents the sum of the mass balance between the proportional contributions of three different carbon sources; to achieve mass balance, the relative contributions of the three different sources (a + b + c) is assumed to sum to 1. The decadal source is the mean (± 1σ) $F^{14}C$ content of atmospheric CO_2 between 1950-2023 using the same data as from Eq. 3 (Methods): 1.226 ± 0.216 Fraction Modern. The millennial source is the carbon-weighted mean (± 1σ) age of global mineral soil carbon in the upper 0-30 cm: 1390 ± 310 ^{14}C years or 0.841 ± 0.033 Fraction Modern[16]. The upper 0-30 cm global soil age was used rather than the 0-100 cm or 30-100 cm age profiles to represent a moderate centennial- to millennial-aged soil carbon source that is older than short-term terrestrial carbon but substantially younger than carbon from petrogenic sources. The petrogenic source is assumed to contain no radiocarbon (i.e., 0.0 Fraction Modern), but since an $F^{14}C$ value of 0.0 cannot be included in the *Simmr* model, we set this to 0.0001 ± 0.0001.

The Bayesian isotope mixing model outputs suggests that decadal carbon sources may have accounted for 31-64% (mean = 50%, median = 51%) of emissions, millennial carbon inputs 23-47%, and petrogenic carbon 5-28% (Table S2; where total river vertical CO_2 emissions = 2.0 ± 0.2 Pg C y^{-1}). There is a considerable amount of variability in these estimated proportional contributions (Extended Fig. 6). This variability is to be expected given the diversity of catchments from which samples were collected, the number of potential sources used, and the range of possible contributions which could give rise to the $F^{14}C$ observations. It would be

unconstructive to further constrain the input components of this analysis to reduce the wide range of potential solutions given the diversity of samples in the database; increasing the number of potential carbon sources would only spread the uncertainty across more sources (noting we chose these three sources following conceptual models elsewhere[17,18]), and constraining the existing source $F^{14}C$ ranges would make them potentially less applicable across the type and timing of samples in the database. Despite these limitations, the values this model produces agree with the dual mixing model with Monte Carlo simulations where petrogenic contributions were used as a prior (as discussed in the main text; Table 1), overall supporting the predicted contributions from different aged carbon sources to river carbon emissions.

S4. Upscaling limitations

Our calculations of global river CO_2 emissions from the three different carbon sources are only intended as first order flux estimates (Table 1). We were not able to scale $F^{14}C$ values with concentration or CO_2 emission flux when calculating the emission of different carbon sources to the atmosphere from global rivers. This was a deliberate decision based on the lack of a clear relationship between $F^{14}C_{atm}$ -DIC and DIC concentrations where paired data was available (Fig. S9A) and a lack of in-situ paired CO_2 flux measurements across the large dataset. DIC concentrations in the database did not scale well with pH either, although higher concentrations of DIC tended to occur at higher pH (Fig. S9B), which is expected following carbonate equilibria (Eq. S2). Where pH values are lower, CO_2 concentrations are likely to be higher based on the pH dependent speciation of DIC. While we show only a small average offset (0.02) between $F^{14}C$-CO_2 and $F^{14}C$-DIC paired measurements (Supplementary Information S2), at lower pH there are some larger differences where $F^{14}C$-CO_2 tends to be ^{14}C-enriched compared to $F^{14}C$ -DIC. Therefore, we note that an important research gap remains as to how the trade off between DIC and CO_2 equilibrium relates to the relative magnitude of emissions from carbon sources across rivers of different biomes, lithologies and catchment size. Further research would also benefit from a combined approach to determine weathering inputs at the small catchment scale in combination with well constrained CO_2 emissions fluxes to enable upscaling of global river CO_2 emissions based on the important lithological controls we describe here. Matching the scales at which we measure weathering rates, the respiration of old soil carbon and river CO_2 emissions would be an important component of this future work.

It is possible that the declining trend of $F^{14}C_{atm}$ in river DIC (Extended Data Fig. 1) could be partially due to studies specifically looking for the presence of old carbon. For example, exploring the role of chemical weathering on river DIC fluxes[3,19], or the release of old carbon due to landscape disturbance[20] or permafrost thaw[21]. Searching the article titles in our database for terms including "peat", "permafrost", "glacier/glacial" and "weathering" returned 282 instances, or ~24% of the database, suggesting there is limited bias towards studies specifically targeting old carbon. Our results implicate carbon of all ages as important contributions to global river emissions; as such future studies could consider systems which may receive carbon of a wide range of ages when seeking to tease apart key processes driving this flux (Extended Data Fig. 7).

We also note that $F^{14}C$-CH_4 observations were very limited in our database, especially after removing repeated sampling locations (n = 19; Fig. S5). As a result, we primarily focused on DIC and CO_2 in this analysis. River $F^{14}C$-CH_4 represents a clear research gap, for both

dissolved and bubble emissions[22]; a suite of methods now exist to collect $F^{14}C-CH_4$ samples across a wide range of aquatic systems to address this gap[23–27].

Future work should also focus on undertaking more river carbon emissions $F^{14}C$ measurements in river systems neglected in our compilation, namely Greenland, Africa, the Arctic and Boreal zones, the Middle East, eastern Europe, western Russia, Central Asia, Australasia, and South America outside of the Amazon (Fig. 1A). Future work could also benefit from investigations into the importance of temporal scales on river $F^{14}C$ emissions, including diurnal, seasonal and long-term patterns, and the importance of hydrologic flow path variability during wet and dry periods and concurrent changes in the magnitude and source of CO_2 and CH_4 fluxes to the atmosphere[28].

Supplementary Figures

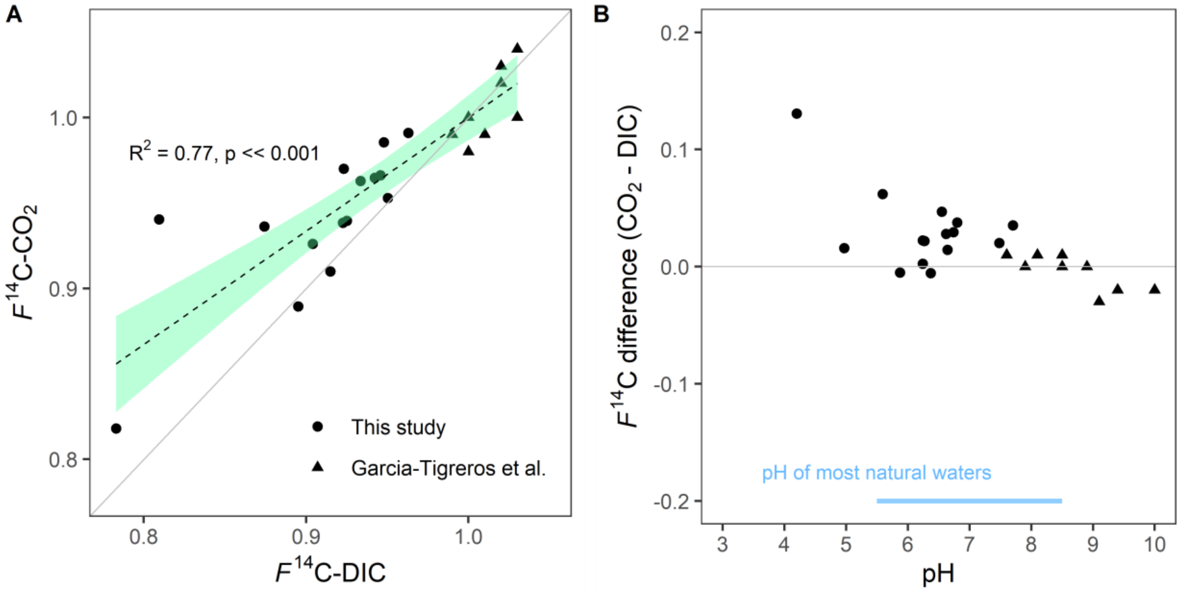

Fig. S1 | CO_2 and DIC $F^{14}C$ equilibrium. (A) Paired CO_2 and DIC $F^{14}C$ values where available from this study (rivers) and ref[12] (Arctic and boreal lakes) – the grey diagonal line indicates a 1:1 relationship; dashed line, R^2 and p-values are from linear regression. (B) The difference in $F^{14}C$ content of paired CO_2 and DIC values over the pH range of natural waters – the grey horizontal line indicates no difference.

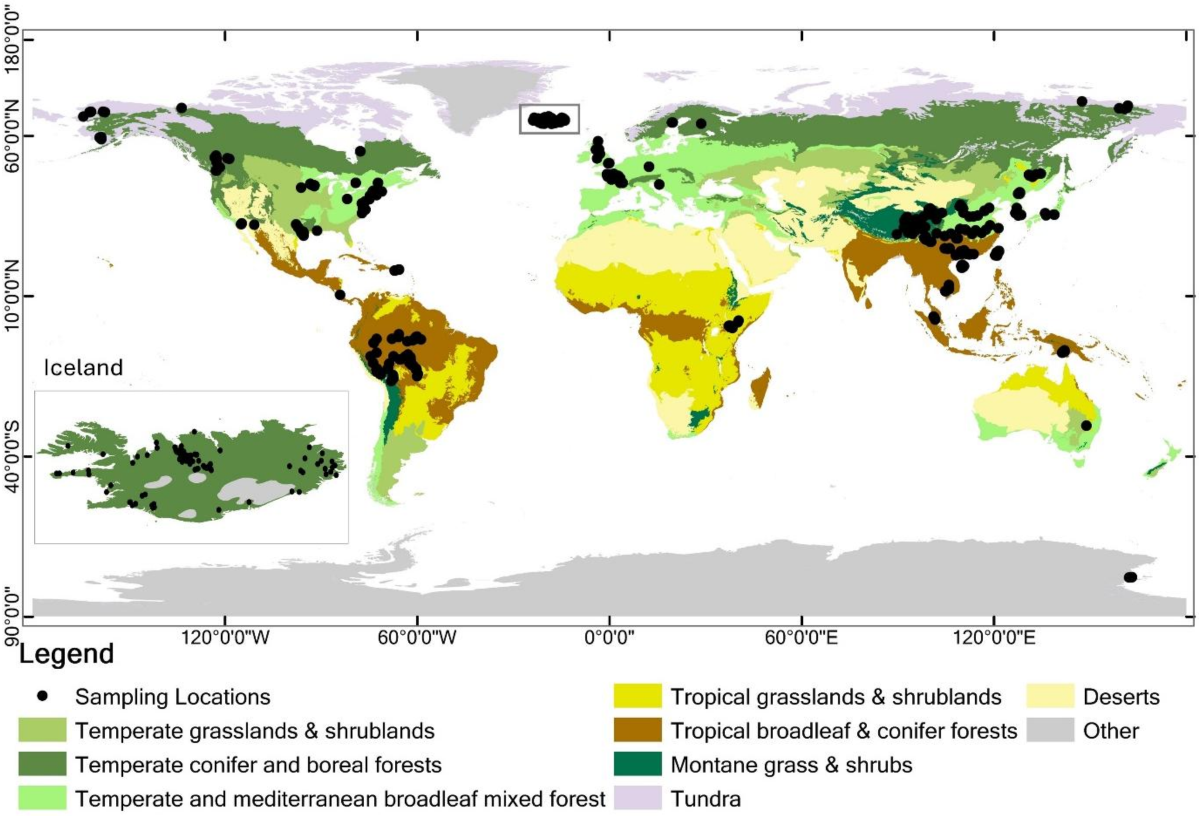

Fig. S2 | Sampling locations and catchment biomes. Locations of the sampling points assembled in the ^{14}C database in the context of catchment biome; the inset shows Iceland where there was a high density of sampling sites. Biomes are adapted from HydroATLAS[29] (Methods).

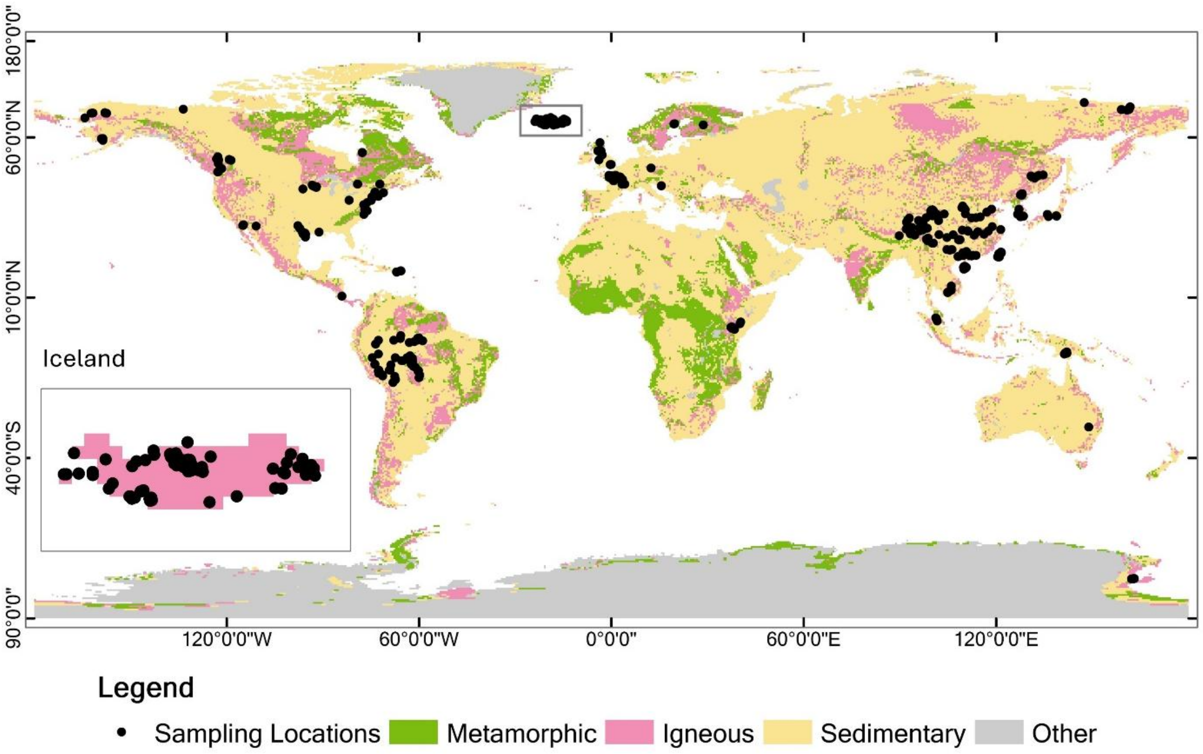

Fig. S3 | Sampling locations and catchment lithology. Locations of the sampling points assembled in the ^{14}C database in the context of catchment lithology; the inset shows Iceland where there was a high density of sampling sites. Lithology classes are adapted from HydroATLAS[29] (Methods).

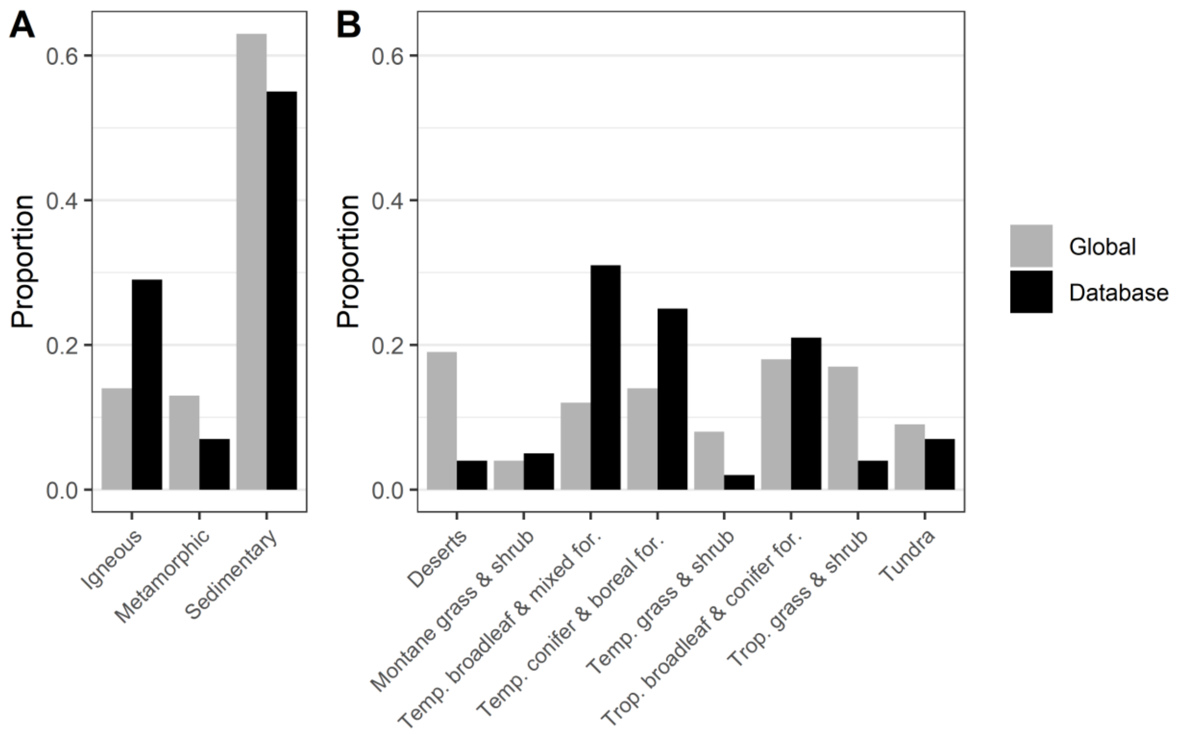

Fig. S4 | Global representativeness of the $F^{14}C$ database sampling locations. Proportion of the global landmass and proportion of the sampling locations in the assembled database underlain by the lithologies (A) and the biomes (B) we define in this study (Methods).

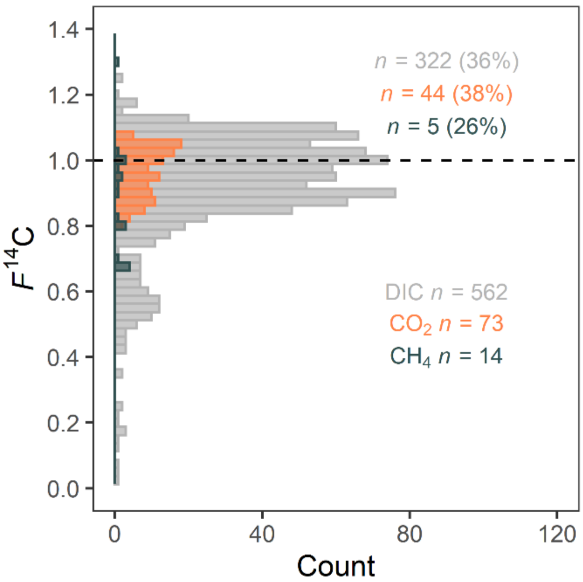

Fig. S5 | River $F^{14}C$ values for DIC, CO_2 and CH_4 having removed repeated samples. Histogram of database $F^{14}C$ observations after averaging values collected from repeated sampling locations (n > 4 in a calendar year; Methods) – the dashed vertical line shows $F^{14}C$ = 1, observation *n* is shown by compound where values fall above or below $F^{14}C$ = 1, values in parentheses indicate percentage observations where $F^{14}C$ > 1.

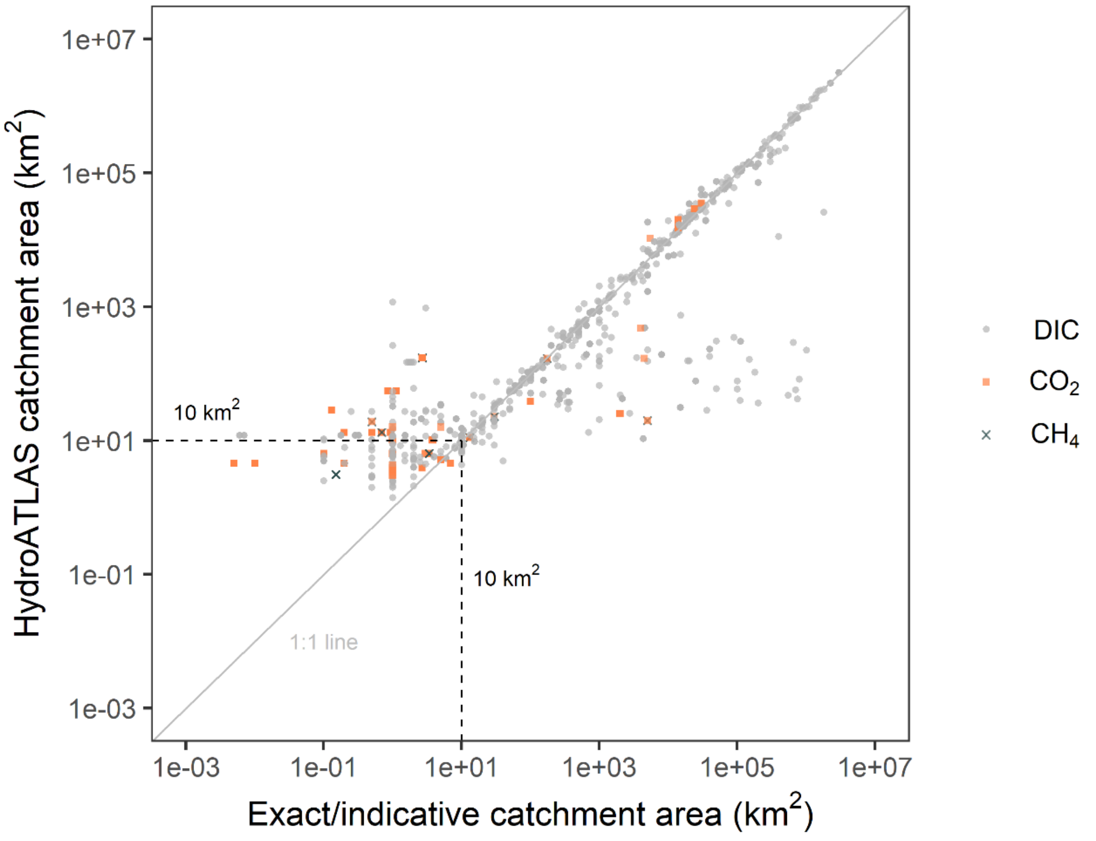

Fig. S6 | Catchment size quality control for HydroATLAS data extraction. Comparison between catchment size generated from HydroATLAS[29] and the exact or indicative catchment size determined for each $F^{14}C$ observation in the database (Methods).

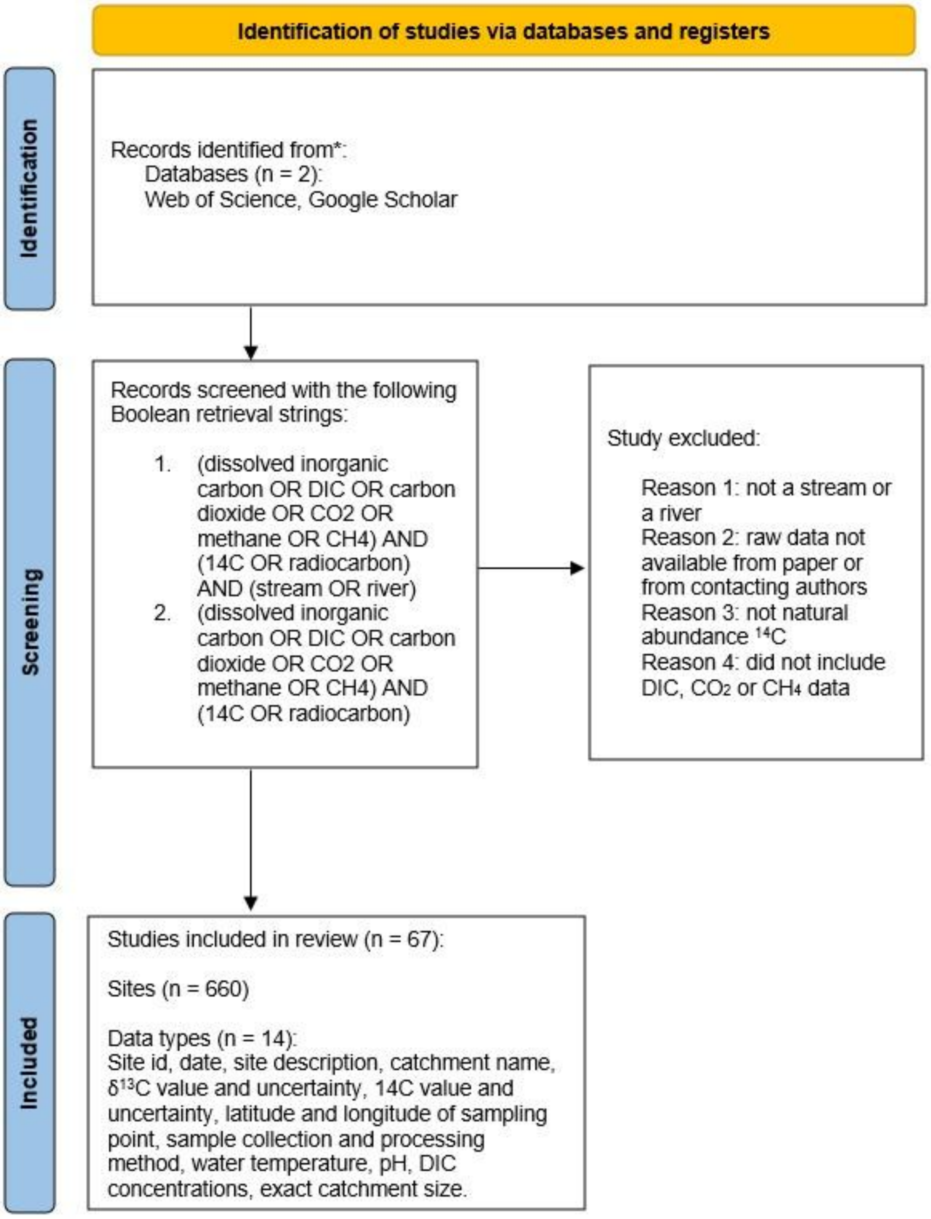

Fig. S7 | Flow diagram of data collection from existing literature. Based on the PRISMA reporting guidelines[30].

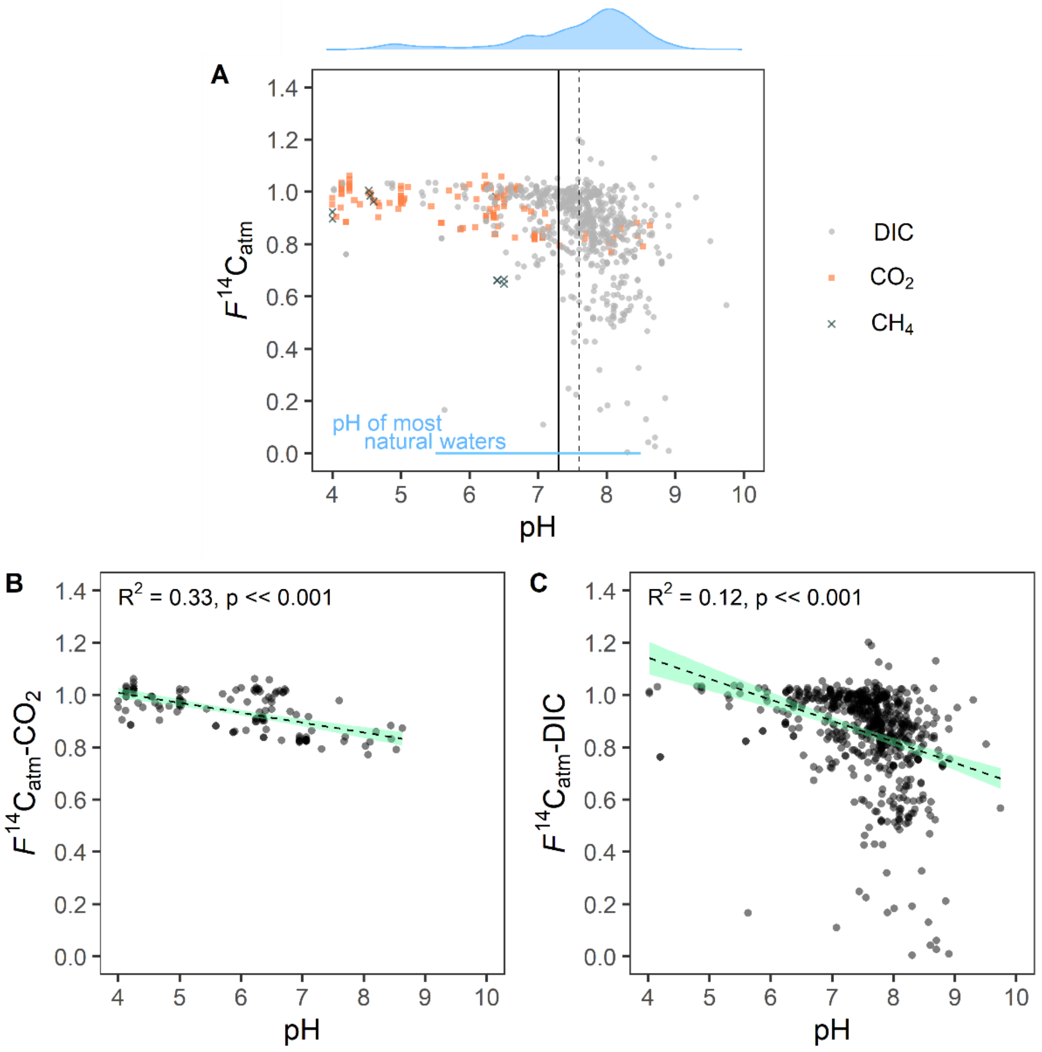

Fig. S8 | $F^{14}C_{atm}$ relationships with pH. (A) $F^{14}C_{atm}$ relationship with pH for all data where available – the blue bar shows the pH range of most natural waters, the vertical solid line indicates the mean pH, the vertical dashed line indicates the median; the density plot for the pH values is shown above the panel. (B) $F^{14}C_{atm}$ relationship with pH for CO_2, only. (C) $F^{14}C_{atm}$ relationship with pH for DIC, only. The dashed line, R^2 and p-values for panels (B) and (C) are from linear regression.

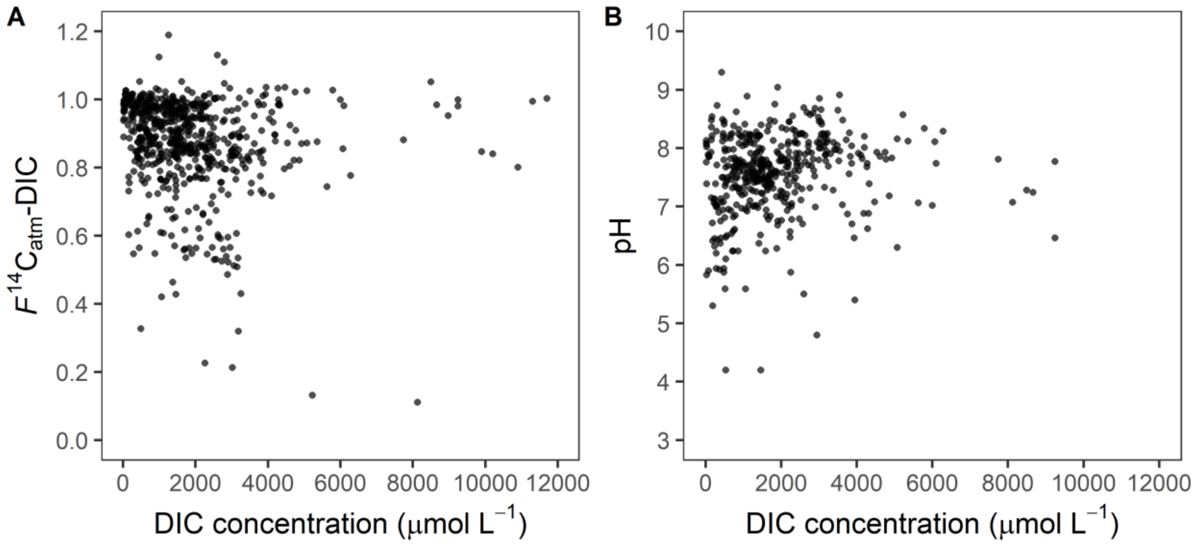

Fig. S9 | DIC concentration relationships with F^{14}C and pH. DIC concentration relationships shown with (A) $F^{14}C_{atm}$-DIC and (B) pH of the river sampling locations.

Supplementary Tables

Supplementary Table S1 | Global database of ^{14}C in river DIC, CO_2 and CH_4. The full database assembled for the analysis presented in this manuscript, including the data collected from the literature, the new data presented here, and the data extracted from HydroATLAS; details of the extracted HydroATLAS parameters are provided in Table S5.

[*included as a separate .xlsx file*]

Supplementary Table S2 | Proportional contributions of carbon sources to global river CO_2 emissions from the Bayesian Isotope mixing model. Proportional contributions for each carbon source estimated from the Bayesian isotope mixing model (Extended Data Fig. 6) are shown for decadal (based on bomb peak atmospheric $^{14}CO_2$; Fig. 1C), millennial (based on ^{14}C content of the top 0-30cm of soils, globally[16]), petrogenic (radiocarbon-dead carbon derived from weathering), and "old" (combined millennial and petrogenic) carbon sources. The proportions are used to estimate the emissions flux of carbon from each of these sources to the atmosphere from global river surfaces[31,32].

	**Decadal**		**Millennial**		**Petrogenic**	
	mean	1σ	mean	1σ	mean	1σ
**Biome (observation *n*)**						
Deserts (37)	0.60	0.13	0.26	0.17	0.13	0.05
Montane grass & shrub (56)	0.31	0.16	0.41	0.23	0.28	0.08
Temp. broadleaf & mixed for. (312)	0.47	0.10	0.40	0.15	0.13	0.05
Temp. conifer & boreal for. (259)	0.64	0.10	0.23	0.14	0.14	0.05
Temp. grass & shrub (24)	0.62	0.10	0.27	0.13	0.11	0.05
Trop. Broadleaf & conifer for. (218)	0.50	0.14	0.36	0.21	0.15	0.06
Trop. grass & shrub (45)	0.32	0.17	0.47	0.25	0.21	0.09
Tundra (69)	0.53	0.09	0.36	0.13	0.11	0.04
**Lithology (observation *n*)**						
Igneous (319)	0.612	0.112	0.25	0.162	0.137	0.051
Metamorphic (73)	0.533	0.046	0.421	0.064	0.046	0.022
Sedimentary (612)	0.486	0.164	0.342	0.238	0.172	0.075

**Combined outputs from Biome & Lithology grouping model runs**	*Decadal*	*Millennial*	*Petrogenic*	*"Old" sources*
minimum	0.31	0.23	0.05	0.28
maximum	0.64	0.47	0.28	0.75
mean	0.50	0.34	0.15	0.49
median	0.51	0.36	0.14	0.49
1σ	0.12	0.17	0.06	0.18
**CO_2 flux to the atmosphere (Pg C y^{-1})**				
Total flux to the atmosphere from global river surfaces	*Decadal*	*Millennial*	*Petrogenic*	*"Old" sources*
2.0 ± 0.2	1.0 ± 0.2	0.7 ± 0.3	0.3 ± 0.1	1.0 ± 0.4

Supplementary Table S3 | Paired $F^{14}C$ measurements of CO_2 and DIC. Sample information on the paired $F^{14}C$ measurements collected concurrently for CO_2 and DIC in Scotland and Peru.

[*included as a separate .xlsx file*]

Supplementary Table S4 | Parameters extracted from HydroATLAS and included in our database. The list of parameters and their descriptions, and whether they were extracted at the reach (R) and/or catchment scale (C) – this was dependent on availability in HydroATLAS.

Parameter code	Description	C	R
dis_m3_pyr	discharge, at sub-basin pour point, annual average in m^3	Y	
lka_pc_use	percent lake area, in total watershed upstream of sub-basin pour point, spatial extent % multiplied by 10	Y	
gwt_cm_cav	groundwater table depth, in sub-basin (reach) only, centimetres		Y
ele_mt_uav	elevation, in total watershed upstream of sub-basin pour point, average meters above sea level (a.s.l.)	Y	Y
slp_dg_uav	terrain slope, in total watershed upstream of sub-basin pour point, average in degree multiplied by 10	Y	
tmp_dc_uyr	air temperature, in total watershed upstream of sub-basin pour point, annual average in degree Celsius multiplied by 10	Y	Y
pre_mm_uyr	precipitation, in total watershed upstream of sub-basin pour point, annual average in mm	Y	Y
ari_ix_uav	aridity index, in total watershed upstream of sub-basin pour point, index value multiplied by 100	Y	Y
snw_pc_uyr	snow cover extent, in total watershed upstream of sub-basin pour point, percent cover %	Y	Y
wet_pc_ug2	wetland class extent, all wetland classes excluding lakes, reservoirs and rivers, percent cover %	Y	Y
for_pc_use	forest cover extent, in total watershed upstream of sub-basin pour point, percent cover	Y	Y
crp_pc_use	cropland extent, in total watershed upstream of sub-basin pour point, percent cover	Y	Y
pst_pc_use	pasture extent, in total watershed upstream of sub-basin pour point, percent cover	Y	Y
ire_pc_use	irrigation area extent (extent of area equipped for irrigation), in total watershed upstream of sub-basin pour point, percent cover	Y	Y
gla_pc_use	glacier extent, in total watershed upstream of sub-basin pour point, percent cover	Y	Y
prm_pc_use	permafrost extent, in total watershed upstream of sub-basin pour point, percent cover	Y	Y
tbi_cl_cmj	terrestrial biomes, in sub-basin only, spatial majority class		Y
fmh_cl_cmj	freshwater major habitat types, in sub-basin only, spatial majority class		Y
cly_pc_uav	clay fraction in soil, at 6 standard depths, in total watershed upstream of sub-basin pour point, 1 km^2 resolution in percent	Y	Y
slt_pc_uav	silt fraction in soil, at 6 standard depths, in total watershed upstream of sub-basin pour point, 1 km^2 resolution in percent	Y	Y
snd_pc_uav	sand fraction in soil, at 6 standard depths, in total watershed upstream of sub-basin pour point, 1 km^2 resolution in percent	Y	Y
soc_th_uav	organic carbon content in soil, at 6 standard depths, in total watershed upstream of sub-basin pour point, 1 km^2 resolution in percent	Y	Y
lit_cl_cmj	lithological classes, in sub-basin, spatial majority		Y
kar_pc_use	karst area extent, in total watershed upstream of sub-basin pour point, percent cover	Y	Y
ero_kh_uav	soil erosion, in total watershed upstream of sub-basin pour point, kg/ha per year	Y	Y
urb_pc_use	urban extent, in total watershed upstream of sub-basin pour point, percent cover	Y	Y
hft_ix_u09	human footprint influence, in total watershed upstream of sub-basin pour point, index value multiplied by 10	Y	Y

Table S5 | Parameters extracted from HydroATLAS and used in the random forest model. The list of parameter codes used for the two model runs based on catchment size – parameter descriptions can be found in Table S4; labels represent what is shown in the results figures.

Small catchments (≤ 10 km^2)		Large catchments (> 10 km^2)	
**Parameter code**	**Label in Extended Data Fig. 4**	**Parameter code**	**Label in Extended Data Fig. 3**
year	Year	year	Year
ele_mt_cav	Mean elevation	catchment_area_km2	Catchment area
pre_mm_cyr	Mean annual precipitation	lka_pc_use	Percent lake area
tmp_dc_cyr	Mean annual temperature	ele_mt_uav	Mean elevation
snw_pc_cyr	Snow cover extent	pre_mm_uyr	Mean annual precipitation
wet_pc_cg2	Wetland class extent	tmp_dc_uyr	Mean annual temperature
for_pc_cse	Forest cover extent	snw_pc_uyr	Snow cover extent
ire_pc_cse	Irrigation area extent	wet_pc_ug2	Wetland class extent
gla_pc_cse	Glacier extent	for_pc_use	Forest cover extent
prm_pc_cse	Permafrost extent	ire_pc_use	Irrigation area extent
cly_pc_cav	Soil clay fraction	gla_pc_use	Glacier extent
snd_pc_cav	Soil sand fraction	prm_pc_use	Permafrost extent
soc_th_cav	Organic carbon content	cly_pc_uav	Soil clay fraction
kar_pc_cse	Karst area extent	snd_pc_uav	Soil sand fraction
ero_kh_cav	Soil erosion	soc_th_uav	Organic carbon content
urb_pc_cse	Urban extent	kar_pc_use	Karst area extent
hft_ix_c09	Human footprint influence	ero_kh_uav	Soil erosion
gwt_cm_cav	Groundwater table depth	hft_ix_u09	Human footprint influence
		gwt_cm_cav	Groundwater table depth

References

1. Soulet, G., Skinner, L. C., Beaupré, S. R. & Galy, V. A Note on Reporting of Reservoir ^{14}C Disequilibria and Age Offsets. *Radiocarbon* 58, 205–211 (2016).
2. Marwick, T. R. *et al.* The age of river-transported carbon: A global perspective. *Global Biogeochem Cycles* 29, 122–137 (2015).
3. Dasari, S., Garnett, M. H. & Hilton, R. G. Leakage of old carbon dioxide from a major river system in the Canadian Arctic. *PNAS Nexus* 3, pgae134 (2024).
4. Stuiver, M. & Polach, H. A. Discussion Reporting of ^{14}C Data. *Radiocarbon* 19, 355–363 (1977).
5. Lewis, E. & Wallace, D. , W. , R. ,. Program Developed for CO_2 System Calculations. Preprint at (1998).
6. Johnson, K. S. Carbon dioxide hydration and dehydration kinetics in seawater1. *Limnol Oceanogr* 27, 849–855 (1982).
7. Zhang, J., Quay, P. D. & Wilbur, D. O. Carbon isotope fractionation during gas-water exchange and dissolution of CO2. *Geochim Cosmochim Acta* 59, 107–114 (1995).
8. Collins, E. L. *et al.* Global patterns in river water storage dependent on residence time. *Nat Geosci* 17, 433–439 (2024).
9. Szaran, J. Achievement of carbon isotope equilibrium in the system HCO_3^- (solution)-CO_2(gas). *Chem Geol* 142, 79–86 (1997).
10. Winnick, M. J. & Saccardi, B. Impacts of Carbonate Buffering on Atmospheric Equilibration of CO_2 , $\delta^{13}C$ DIC , and $\Delta^{14}C$ DIC in Rivers and Streams. *Global Biogeochem Cycles* 38, (2024).

11. Bourke, S. A., Harrington, G. A., Cook, P. G., Post, V. E. & Dogramaci, S. Carbon-14 in streams as a tracer of discharging groundwater. *J Hydrol (Amst)* 519, 117–130 (2014).
12. Garcia-Tigreros, F. *et al.* Arctic-boreal lakes of interior Alaska dominated by contemporary carbon. *Environmental Research Letters* 18, 124024 (2023).
13. Garnett, M. H., Billett, M. F. F., Gulliver, P. & Dean, J. F. A new field approach for the collection of samples for aquatic $^{14}CO_2$ analysis using headspace equilibration and molecular sieve traps: the super headspace method. *Ecohydrology* 9, 1630–1638 (2016).
14. Dean, J. F. *et al.* East Siberian Arctic inland waters emit mostly contemporary carbon. *Nat Commun* 11, 1627 (2020).
15. Parnell, A. C. *et al.* Bayesian stable isotope mixing models. *Environmetrics* 24, 387–399 (2013).
16. Shi, Z. *et al.* The age distribution of global soil carbon inferred from radiocarbon measurements. *Nat Geosci* 13, 555–559 (2020).
17. Butman, D. E., Wilson, H. F., Barnes, R. T., Xenopoulos, M. A. & Raymond, P. A. Increased mobilization of aged carbon to rivers by human disturbance. *Nat Geosci* 8, 112–116 (2015).
18. Casas-Ruiz, J. P. *et al.* Integrating terrestrial and aquatic ecosystems to constrain estimates of land-atmosphere carbon exchange. *Nat Commun* 14, 1571 (2023).
19. Sveinbjörnsdóttir, Á. E. *et al.* Assessing the sources of inorganic carbon in surface-, soil- and non-thermal groundwater in Iceland by $\delta^{13}C$ and ^{14}C. *Geochim Cosmochim Acta* 279, 165–188 (2020).
20. Moore, S. *et al.* Deep instability of deforested tropical peatlands revealed by fluvial organic carbon fluxes. *Nature* 493, 660–663 (2013).
21. Drake, T. W. *et al.* The Ephemeral Signature of Permafrost Carbon in an Arctic Fluvial Network. *J Geophys Res Biogeosci* 123, 1475–1485 (2018).
22. Rocher-Ros, G. *et al.* Global methane emissions from rivers and streams. *Nature* 621, 530–535 (2023).
23. Dean, J. F., Billett, M. F., Murray, C. & Garnett, M. H. Ancient dissolved methane in inland waters revealed by a new collection method at low field concentrations for radiocarbon (^{14}C) analysis. *Water Res* 115, 236–244 (2017).
24. Elder, C. D. *et al.* Greenhouse gas emissions from diverse Arctic Alaskan lakes are dominated by young carbon. *Nat Clim Chang* 8, 166–171 (2018).
25. Garnett, M. H. & Dean, J. F. A Time-Integrated sampler for radiocarbon analysis of aquatic methane. *Radiocarbon* 1–15 (2024) doi:10.1017/RDC.2024.31.
26. Garnett, M. H., Gulliver, P. & Billett, M. F. A rapid method to collect methane from peatland streams for radiocarbon analysis. *Ecohydrology* 9, 113–121 (2016).
27. Sparrow, K. J. *et al.* Limited contribution of ancient methane to surface waters of the U.S. Beaufort Sea shelf. *Sci Adv* 4, (2018).
28. Duvert, C., Butman, D. E., Marx, A., Ribolzi, O. & Hutley, L. B. CO_2 evasion along streams driven by groundwater inputs and geomorphic controls. *Nat Geosci* 11, 813–818 (2018).
29. Linke, S. *et al.* Global hydro-environmental sub-basin and river reach characteristics at high spatial resolution. *Sci Data* 6, 283 (2019).
30. Page, M. J. *et al.* The PRISMA 2020 statement: an updated guideline for reporting systematic reviews. *BMJ* n71 (2021) doi:10.1136/bmj.n71.

31. Liu, S. *et al.* The importance of hydrology in routing terrestrial carbon to the atmosphere via global streams and rivers. *Proceedings of the National Academy of Sciences* 119, (2022).
32. Lauerwald, R. *et al.* Inland Water Greenhouse Gas Budgets for RECCAP2: 1. State-Of-The-Art of Global Scale Assessments. *Global Biogeochem Cycles* 37, (2023).
